# Chest Radiograph Screening for Detecting Subclinical Tuberculosis in Asymptomatic Household Contacts, Peru

**DOI:** 10.3201/eid3006.231699

**Published:** 2024-06

**Authors:** Qi Tan, Chuan-Chin Huang, Mercedes C. Becerra, Roger Calderon, Carmen Contreras, Leonid Lecca, Judith Jimenez, Rosa Yataco, Jerome T. Galea, Jia-Yih Feng, Sheng-Wei Pan, Yen-Han Tseng, Jhong-Ru Huang, Zibiao Zhang, Megan B. Murray

**Affiliations:** Harvard Medical School, Boston, Massachusetts, USA (Q. Tan, C.-C. Huang, M.C. Becerra, M.B. Murray);; Brigham and Women’s Hospital, Boston (C.-C. Huang, M.C. Becerra, Z. Zhang, M.B. Murray);; Partners In Health—Socios En Salud Sucursal Peru, Lima, Peru (R. Calderon, C. Contreras, L. Lecca, J. Jimenez, R. Yataco);; University of South Florida, Tampa, Florida, USA (J.T. Galea);; Taipei Veterans General Hospital, Taipei, Taiwan (J.-Y. Feng, S.-W. Pan, Y.-H. Tseng, J.-R. Huang);; National Yang Ming Chiao Tung University, Taipei (J.-Y. Feng, S.-W. Pan, Y.-H. Tseng, J.-R. Huang);; Harvard T.H. Chan School of Public Health, Boston (M.B. Murray)

**Keywords:** tuberculosis and other mycobacteria, bacteria, subclinical infections, pulmonary, mass chest x-ray, chest radiography, contact tracing, global health, Peru

## Abstract

The World Health Organization’s end TB strategy promotes the use of symptom and chest radiograph screening for tuberculosis (TB) disease. However, asymptomatic early states of TB beyond latent TB infection and active disease can go unrecognized using current screening criteria. We conducted a longitudinal cohort study enrolling household contacts initially free of TB disease and followed them for the occurrence of incident TB over 1 year. Among 1,747 screened contacts, 27 (52%) of the 52 persons in whom TB subsequently developed during follow-up had a baseline abnormal radiograph. Of contacts without TB symptoms, persons with an abnormal radiograph were at higher risk for subsequent TB than persons with an unremarkable radiograph (adjusted hazard ratio 15.62 [95% CI 7.74–31.54]). In young adults, we found a strong linear relationship between radiograph severity and time to TB diagnosis. Our findings suggest chest radiograph screening can extend to detecting early TB states, thereby enabling timely intervention.

Recognizing the key role early detection plays in interrupting further tuberculosis (TB) transmission, the World Health Organization (WHO) has emphasized the need for global investment in that area. In line with that objective, the end TB strategy promotes the use of TB symptom and chest radiograph screening for active case finding (*1*).

Chest radiography has been widely used as a diagnostic and screening tool for TB (*2*,*3*). Currently, the impact of chest radiograph screening largely depends on its ability to detect patients with clinically apparent TB disease (*4*–*6*). Recent understanding of TB has revealed a heterogeneous period of pathophysiological transition with a spectrum of early TB states, ranging from the time of exposure to *Mycobacterium tuberculosis* to the onset of symptomatic active disease (*7*–*10*). However, asymptomatic early states of TB beyond latent and active disease can be potentially unrecognized using current diagnostic or screening criteria for TB (*11*–*14*).

Accessing chest radiograph screening in low-resource settings, particularly in low- and middle-income countries, has been challenging and has hindered early detection of disease. However, WHO recommended chest radiography with computer-aided detection as a screening tool for TB in its 2021 guidelines (*15*), holding promise for the early detection and triage of TB.

In this study, we used data from a longitudinal cohort of household contacts of TB patients in Lima, Peru, to examine the frequency of baseline abnormal chest radiographs (defined as the presence of any intrathoracic abnormalities compatible with TB disease) in adult contacts who were initially classified as free from TB disease and in whom incident disease subsequently developed. We further evaluated the association between severity of radiograph abnormalities and time to occurrence of TB disease. Our objective was to assess whether baseline chest radiograph screening can identify early TB states among asymptomatic household contacts beyond active case finding.

## Methods

### Study Design and Participants

During September 1, 2009–August 29, 2012, we conducted a prospective longitudinal cohort study of household contacts of index TB patients in Lima, encompassing 20 districts and ≈3.3 million residents of urban areas and peri-urban, informal shantytown settlements. Peru is a middle-income country with TB services concentrated in district health centers. Chest radiograph was not recommended by Peru’s National TB Program Guidelines for community screening during the cohort study period. In the parent study, we enrolled 4,500 index patients >15 years of age with newly diagnosed pulmonary TB from 106 district health centers in Lima (*16*). The study was approved by the Institutional Review Board of Harvard School of Public Health and the Research Ethics Committee of the National Institute of Health of Peru. All study participants provided informed consent.

### Procedures

**Figure 1:**
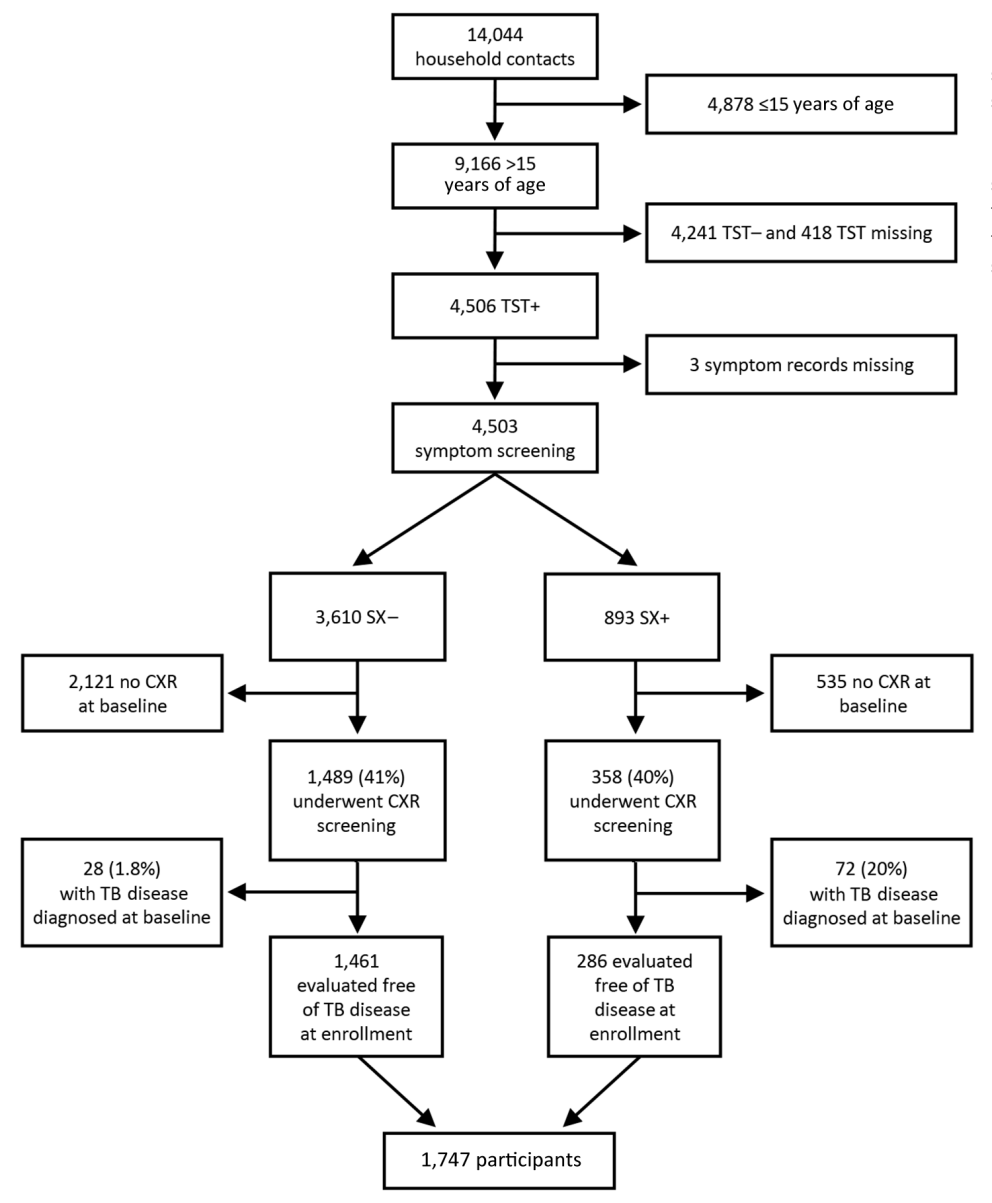
Flowchart of enrollment in study of chest radiograph screening for detection of subclinical TB in asymptomatic household contacts, Peru. CXR, chest radiograph; SX–, no symptoms; SX+, symptoms; TB, tuberculosis; TST–, tuberculin skin test negative; TST+, tuberculin skin test positive.

In the parent study, health center clinicians diagnosed pulmonary TB according to Peru’s National TB program guidelines (*17*), which required >1 of 2 sputum smears to be positive for acid-fast bacilli by Ziehl-Neelsen staining or, in the absence of positive sputum smears, a chest radiograph consistent with TB. We then visited patient households to enroll all household contacts. We gave contacts with no history of TB infection or disease a tuberculin skin test (TST). Health center clinicians made the decision to request a baseline radiograph in symptomatic household contacts. To elucidate why clinicians ordered those radiographs, we compared baseline clinical characteristics in contacts who received a radiograph and those who did not. We screened all enrolled contacts for TB symptoms (cough for >14 days, coughing blood or phlegm, fever, shortness of breath, or night sweats). We used a standardized case report form to collect symptoms; that information was obtained by Spanish or Quechua speakers in private in health clinics or in persons’ homes. Household contacts chosen for evaluation by health center clinicians provided 2 sputum samples for smear microscopy and culture on solid media and were referred for a routine work-up that included a chest radiograph. Persons who did not receive a TB diagnosis at enrollment were then followed for the occurrence of TB disease over a 12-month period. Those in whom incident disease developed were given TB treatment according to Peru’s National TB guidelines (*17*). We collected demographic and health data for each enrolled contact consisting of height and weight; previous TB history; smoking or drinking addiction status; and any comorbidity of HIV infection, diabetes, hypertension, cardiovascular disease, kidney disease, or asthma. We limited our analysis to persons >15 years of age who were TST-positive (Table 1) and who did not receive a diagnosis of TB disease within the first 30 days after enrollment (Figure 1).

**Table 1:** Baseline characteristics of adult tuberculin skin test–positive household contacts of TB patients, Peru*

Characteristic	No. (%)
Age group. y	
16–24	985 (22)
25–44	1,879 (42)
>45	1,642 (36)
Total	4,506 (100)
Sex	
F	2,624 (58)
M	1,882 (42)
BCG-vaccinated	4,070 (90)
History of TB	1,017 (22)
HIV-positive	27 (1)
Diabetes mellitus	131 (3)
With no TB symptoms	3,610 (80)
Baseline chest radiograph performed	1,848 (41)
TB diagnosis received at baseline	252 (6)

*BCG, Bacillus Calmette–Guérin; TB, tuberculosis.

### Radiograph Evaluation and Study Outcomes

We retrospectively evaluated chest radiographs of participants who received a radiograph within 10 days of enrollment. The digitalized anteroposterior films were reevaluated by 3 readers. The first and second readers were both senior pulmonologists with 10–15 years of experience in TB care and TB-related radiographic evaluation. The third reader was a senior radiologist specializing in TB with 20 years’ experience in chest radiologic evaluation for TB diagnosis. Radiologic findings and scoring were assessed following a well-established radiologic protocol (*4*,*17*–*20*) (Appendix). That protocol outlines 3 parameters: whether the radiograph is abnormal, whether abnormalities are suggestive of TB, and the extent of abnormalities (Appendix Table 2). The first reader was blinded to symptoms, TST results, and clinical outcomes. The second reader reviewed chest radiographs deemed abnormal by the first reader to validate those readings. Discrepant interpretations were resolved by a third reader who adjudicated on the basis of the protocol. The final scores for each radiograph were calculated by averaging the results from the 2 readers. Final decisions were reached by consensus. We considered a radiograph to be abnormal at enrollment if any intrathoracic abnormalities were present including cavitation, noncavitary lung lesions, hilar lymphadenopathy, and pleural disorders (Appendix). We classified a parenchymal lesion as noncavitary if we found patchy or confluent consolidation, ground glass opacity, noncalcified nodules, calcified nodules (Ghon focus), diffuse micronodules (miliary pattern), fibrosis, bronchiectasis, collapse (atelectasis), and hyperinflation (*18*,*19*). We further categorized those abnormal findings as TB-suggestive or TB-nonsuggestive (Appendix). To grade the extent of abnormalities, we reported the percentage of lung affected by any pathological abnormality for each of 3 zones (upper, middle, and lower) in each lung. We estimated the total percentage of lung affected by summing the percentages of the 6 zones (0–6) (Appendix) (*18*). We defined the study outcome as incident TB disease detected during a 12-month follow-up period in participants in whom TB disease had been ruled out at baseline.

### Statistical Analyses

#### Associations between Baseline Abnormalities and Incident TB Disease

We used a Cox proportional hazards model to investigate the association between baseline chest radiograph abnormalities and the occurrence of incident TB disease. We categorized participants into 4 groups: those with no TB symptoms and normal chest radiograph (SX–CXR– group); those with TB symptoms and normal radiograph (SX+CXR– group); those with no TB symptoms and abnormal radiograph (SX–CXR+ group); and those with TB symptoms and abnormal radiograph (SX+CXR+ group). We first performed a univariate analysis, followed by a multivariate model that adjusted for potential confounders: age, sex, alcohol use, tobacco use, diabetes, TB history, hypertension, cardiovascular disease, kidney disease, asthma, and body mass index (BMI). We considered that older household contacts and those with a history of TB might be more likely to have residual abnormalities on radiographs that were unrelated to recent TB exposure (*21*,*22*). We therefore repeated our analyses in 3 age subgroups: persons 16–24 years of age, 25–44 years of age, and >45 years of age. In a second sensitivity analysis, we restricted our analyses to contacts with no known previous TB history.

#### Association between Severity of Radiograph Abnormality and TB Progression Trajectory

We used linear regression to evaluate the association between the extent of abnormality on baseline radiograph and the trajectory of TB progression, indicated by the time to the occurrence of TB disease, among household contacts in whom incident TB disease developed during the 1-year follow-up period. Similar to the process discussed previously, we repeated the analyses in 3 age groups and among persons with no known previous TB history, conducting those analyses separately.

To determine whether consistent baseline radiograph readings could serve as an indicator of the risk for incident TB, we compared the risk for incident TB among household contacts with concordant versus discordant radiograph readings. We conducted an analysis using the Pearson χ^2^ test to identify any association between the risk for incident TB and radiograph concordance. We performed all statistical analyses using R version 4.2.2 (The R Foundation for Statistical Computing, https://www.r-project.org).

## Results

Among 9,166 adult household contacts, 4,506 (49%) were TST-positive at baseline; of those, 4,503 (99%) underwent symptom screening. Of the 3,610 persons with TB symptoms and 893 without symptoms at baseline, 1,489 (41%) persons with symptoms and 358 (40%) without symptoms underwent a chest radiograph at baseline. Those referred for chest radiograph were less likely to be male (37% vs. 45%; p = 0.001), HIV-positive (0.4% vs. 0.8%; p = 0.02), or have a previous history of TB disease (17% vs. 26%; p<0.001) (Appendix Table 1). The presence of TB symptoms was similar among those referred for radiograph and those not referred (19% vs. 20%; p = 0.54). Of the 1,847 subjects with a baseline radiograph, TB disease developed in 100 (5%) persons within 30 days after enrollment; we considered those to be coprevalent cases and excluded them from analysis (Figure 1).

### Associations between Baseline Radiograph Abnormalities and Incident TB Disease

#### Subsequent TB in All Screened Participants

**Figure 2:**
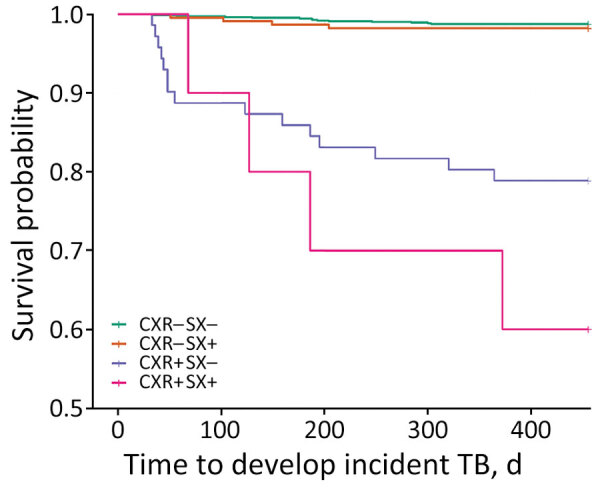
Associations between chest radiograph and symptom screening results and time to incident TB among tuberculin skin test–positive adults, Peru. N = 1,747, incident events = 52. CXR–, unremarkable chest radiograph; CXR+, abnormal chest radiograph; SX–, no symptoms; SX+, symptoms; TB, tuberculosis.

Among the 1,747 persons who were determined to be TB-negative at baseline and who had a baseline radiograph, 52 (3%) received a diagnosis of incident TB disease within the next 12 months. Among those 52 persons, 25 (48%) of the diagnoses were confirmed through microbiological tests and 8% through chest radiograph and symptoms; the remaining 44% lacked diagnostic information. Of the 52 persons, 38 (73%) were asymptomatic at baseline and 27 (52%) had an abnormal chest radiograph result at baseline (Appendix Figure 1). Of the 27 with abnormal radiographs, 2 (7%) had cavities, 18 (67%) had noncavitary parenchymal lesions, 6 (22%) had hilar lymphadenopathy, and 4 (15%) had pleural disorders; 3 (11%) of the 27 radiographs were agreed upon by 2 readers to have >1 abnormality. (Appendix Table 3, Figure 2). The risk for TB disease in those with an abnormal radiograph was higher both among those with initial symptoms and those without symptoms than for persons with normal x-ray and no symptoms at baseline (adjusted hazard ratio [aHR] of SX+CXR– vs. SX–CXR– was 2.24 [95% CI 0.92–5.74]; aHR of SX–CXR+ vs. SX–CXR– was 15.62 [95% CI 7.74–31.54]; aHR of SX+CXR+ vs. SX–CXR– was 26.50 [95% CI 9.98–70.36]) (Table 2; Figure 2). Among the 135 abnormal radiographs evaluated by 2 readers, readers agreed on the presence or absence of abnormalities in 133 (99%) films. The risk for TB disease was higher in contacts in whom readers disagreed on the presence of noncavitary parenchymal lesions (Appendix Table 4).

**Table 2:** Chest radiograph and symptom screening results and their association with risk for subsequent TB in tuberculin skin test–positive adults, Peru*

Result†	No. household contacts	No. (%) incident TB cases	Univariate analysis			Multivariate analysis‡	
			Hazard ratio (95% CI), n = 1,747, events = 52§	p value		Hazard ratio (95% CI), n = 1,630, events = 49§	p value
CXR–SX–	1,349	18 (1)	Referent			Referent	
CXR–SX+	263	7 (3)	2.01 (0.84–4.82)	0.12		2.24 (0.92–5.47)	0.08
CXR+SX–	112	20 (18)	14.64 (7.74–27.67)	<0.001		15.62(7.74–31.54)	<0.001
CXR+SX+	23	7 (30)	26.85 (11.21–64.31)	<0.001		26.50 (9.98–70.36)	<0.001

*In both analyses, n indicates number of analyzed participants and event indicates number of participants in whom incident TB developed. CXR–, unremarkable chest radiograph, CXR+, abnormal chest radiograph; SX–, no symptoms; SX+, symptoms; TB, tuberculosis. †Household contacts were categorized into 4 groups by screening results: CXR–SX–, n = 1,349; CXR–SX+, n = 263; CXR+SX–, n = 112; CXR+SX+, n = 23. Symptoms were defined as any of the following: cough>14 d, cough with blood or phlegm, fever, shortness of breath, night sweats. Abnormal chest radiograph was defined as a radiograph with any intra-thorax abnormalities compatible with TB. ‡Adjusted for age, sex, alcohol use, tobacco use, diabetes, hypertension, cardiovascular disease, kidney disease, asthma, previous TB history, and body mass index. HIV-positive participants (n = 4) were excluded.

#### Subsequent TB by Age Groups

**Figure 3:**
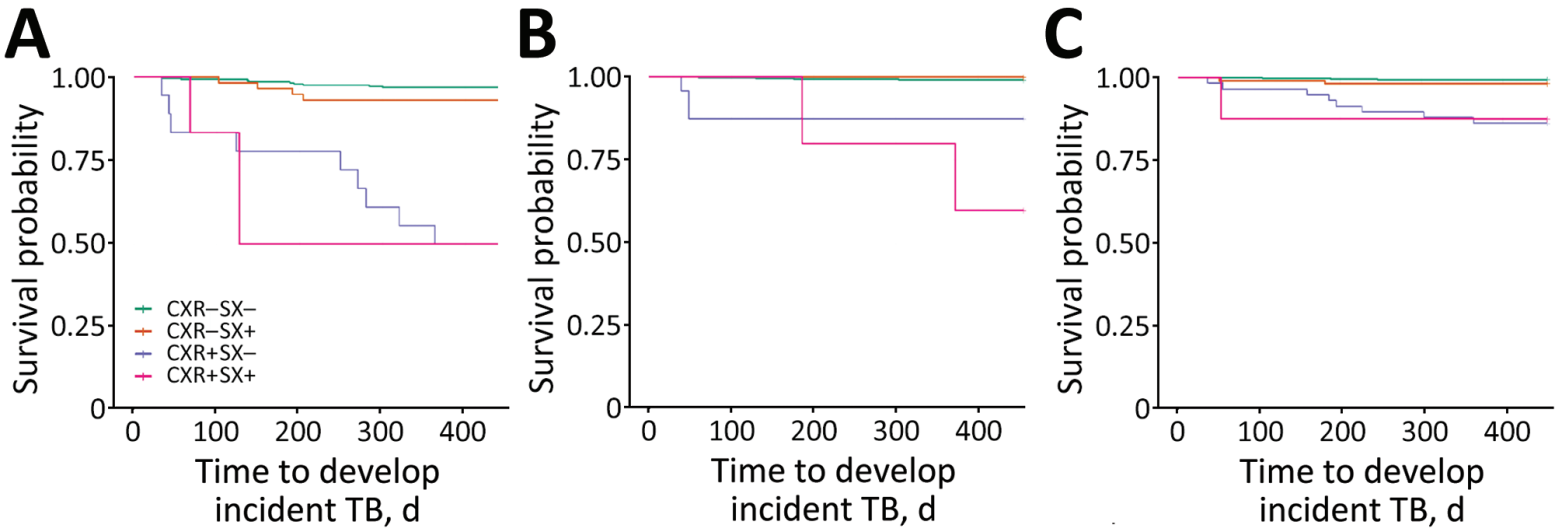
Associations between abnormal chest radiographs and time to incident TB among tuberculin skin test–positive adults in 3 age groups, Peru. A) 16–24-year age group (N = 370, incident events = 25); B) 25–44-year age group (N = 709, incident events = 13); C) >45-year age group (N = 668, incident events = 14). CXR–, unremarkable chest radiograph, CXR+, abnormal chest radiograph; SX–, no symptoms; SX+, symptoms; TB, tuberculosis.

The risk for incident TB disease fell from 7% in participants 16–24 years of age to 2% in persons 25–44 years of age and 2% in those >45 years of age (Appendix Table 5, Figure 3). A similar pattern was observed in participants with an abnormal radiograph at enrollment (Appendix Table 6, Figure 3). Among participants 16–24 years of age with abnormal radiographs at baseline, incident TB developed in 12 (50%) persons; 9 of those (75%) were asymptomatic at enrollment. In contrast, among those with an abnormal radiograph, TB developed in only 6 (13%) of those 25–44 years of age and 9 (14%) of those >45 years of age; of those, 3 (50%) persons 25–44 years of age and 8 (89%) persons >45 years of age were asymptomatic (Appendix Figure 6). The risk for subsequent TB disease was higher in both initially asymptomatic and symptomatic persons of all subgroups who had an abnormal baseline radiograph than in persons with a normal radiograph (Table 3; Figure 3).

**Table 3:** Chest radiograph and symptom screening results and association with risk for subsequent TB, by age group, Peru*

Age group, y†	No. (%) incident TB cases	Univariate analysis			Multivariate analysis	
		Hazard ratio (95% CI)	p value		Hazard ratio (95% CI)	p value
16–24		n = 370, events = 25			n = 360, events = 24‡	
CXR–SX–	9 (0.3)	Referent			Referent	
CXR–SX+	4 (7)	2.25 (0.69–7.31)	0.18		2.12(0.59–7.55)	0.25
CXR+SX–	9 (50)	20.47 (8.11–51.64)	<0.001		19.12 (7.19–50.79)	<0.001
CXR+SX+	3 (50)	24.26 (6.53–90.12)	<0.001		21.03 (5.12–86.30)	<0.001
25–45		n = 709, events = 13			n = 664, events = 13§	
CXR–SX–	6 (1)	Referent			Referent	
CXR–SX+	1 (1)	0.93 (0.11–7.73)	0.95		0.89 (0.11–7.57)	0.92
CXR+SX–	3 (8)	8.38 (2.09–33.51)	<0.01		8.85 (2.09–37.44)	<0.01
CXR+SX+	3 (33)	33.21 (8.29–132.97)	<0.001		28.89 (6.07–137.67)	<0.001
>45		n = 668, events = 14			n = 630, events = 14¶	
CXR–SX–	3 (0.6)	Referent			Referent	
CXR–SX+	2 (2)	3.23 (0.53–19.32)	0.19		3.01 (0.49–18.44)	0.23
CXR+SX–	8 (14)	24.21 (6.42–91.26)	<0.001		18.32 (4.37–76.87)	<0.001
CXR+SX+	1 (12)	22.98 (2.39–220.96)	<0.01		29.22 (2.42–352.91)	<0.001

*In both analyses, n indicates number of analyzed participants and event indicates number of participants in whom incident TB developed. CXR–, unremarkable chest radiograph, CXR+, abnormal chest radiograph; SX–, no symptoms; SX+, symptoms; TB, tuberculosis. †Symptoms were defined as any of the following: cough >14 d, cough with blood or phlegm, fever, shortness of breath, night sweats. Abnormal chest radiograph was defined as a radiograph with any intra-thorax abnormalities compatible with TB. Abnormal CXR was defined as a CXR with any intra-thorax abnormalities compatible with TB. ‡Adjusted for sex, cardiovascular disease, kidney disease, asthma, TB history, and body mass index. All persons in this age group were HIV negative. §Adjusted for sex, alcohol use, hypertension, kidney disease, asthma, TB history, and body mass index. HIV-positive persons (n = 4) were excluded. ¶Adjusted for sex, alcohol use, tobacco use, diabetes, hypertension, TB history, and body mass index. All persons in this age group were HIV negative.

#### Subsequent TB in Participants with No TB History

When we restricted our analysis to the 1,440 persons with no known TB history, the effect sizes for the associations between radiograph status and subsequent incident TB increased by 30% compared with the analysis that included adult contacts with a TB history. aHRs were 23.11 (95% CI 10.35–51.57) for SX–CXR+ versus SX–CXR– and 34.24 (95% CI 9.65–124.54) for SX+CXR+ versus SX–CXR– (Table 4).

**Table 4:** Chest radiograph and symptom screening results and their association with risk for subsequent TB in adults with no known TB history*

Result†	No. (%) incident TB cases	Univariate analysis			Multivariate analysis‡	
		Hazard ratio (95% CI), n = 1,440, event = 37	p value		Hazard ratio (95% CI), n = 1,347, event = 34	p value
CXR–SX–	14 (1)	Referent			Referent	
CXR–SX+	4 (3)	1.45 (0.48–4.39)	0.51		1.64 (0.53–5.11)	0.39
CXR+SX–	15 (21)	19.46 (9.39–40.33)	<0.001		23.11 (10.35–51.57)	<0.001
CXR+SX+	4 (40)	38.53 (12.67–117.18)	<0.001		34.24 (9.65–121.54)	<0.001

*In both analyses, n indicates number of analyzed participants and event indicates number of participants in whom incident TB developed. CXR–, unremarkable chest radiograph, CXR+, abnormal chest radiograph; SX–, no symptoms; SX+, symptoms; TB, tuberculosis. †Symptoms were defined as any of the following: cough>14 d, cough with blood or phlegm, fever, shortness of breath, night sweats. Abnormal chest radiograph was defined as a radiograph with any intra-thorax abnormalities compatible with TB. Abnormal CXR was defined as a CXR with any intra-thorax abnormalities compatible with TB. ‡Adjusted for age, sex, alcohol use, tobacco use, diabetes, hypertension, cardiovascular disease, kidney disease, asthma, TB history and BMI. HIV-positive persons (n = 3) were excluded.

### Associations between Severity of Radiograph Abnormality and TB Progression Trajectory

**Figure 4:**
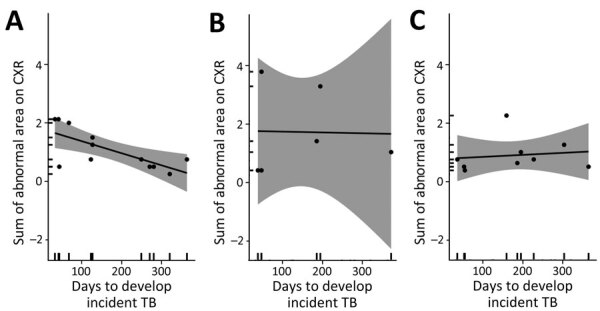
Association between degree of baseline chest radiograph severity and time to developing incident TB among persons with abnormal radiograph findings by age group, Peru. Gray shading indicates 95% CIs. A) 16–24-year age group (n = 12). Mean difference −0.004 (95% CI −0.007 to −0.001); p<0.001, ρ = −0.71; B) 25–44-year age group (n = 6). Mean difference −0.0002 (95% CI −0.015 to 0.015); p = 0.96, ρ = −0.025; C) >45-year age group (n = 9). Mean difference 0.0006 (95% CI −0.004 to 0.005); p = 0.73, ρ = 0.14. ρ, Pearson correlation coefficient.

The severity grade of the baseline radiograph was not significantly associated with time from enrollment to TB diagnosis (mean difference of −0.002 [95% CI –0.005 to 0.001] days; n = 27) among all adults in whom incident TB developed (Appendix Figure 5). However, we found a strong linear relationship between the radiograph severity grade and time to TB diagnosis among participants 16–24 years of age (mean difference of −0.004 [95% CI –0.007 to −0.001]; n = 12) but not among older persons (Figure 4). We also did not find an association between severity grade and time to incident TB among persons with no known TB history (mean difference of 0.002 [95% CI –0.006 to 0.001]; n = 19) (Appendix Figure 6).

## Discussion

In this study, we found that among persons >15 years of age who were exposed to TB at home, those who were initially deemed TB-free but had an abnormal baseline chest radiograph had a 15-fold higher risk for incident TB developing during 12 months of follow-up than did persons with an unremarkable radiograph. That finding was more pronounced in household contacts with no TB history and in younger participants, who were less likely to have residual abnormalities on radiograph from past TB or other comorbidities. Our findings remained unaltered in multivariate models when we further adjusted for demographic and clinical variables, as well as history of comorbidity. Among young persons with abnormal radiograph who were initially deemed TB-free at baseline, incident TB developed in 50% during follow-up. The severity grade of radiograph abnormalities was a strong indicator of an early-state TB trajectory in young persons.

Although numerous investigators have examined the use of chest radiography in screening for clinically apparent TB, few have evaluated whether abnormal radiographs could help identify early TB. The idea of using chest radiograph examination for incipient pulmonary TB was first proposed by Dr. Francis Williams, the father of chest radiology in the United States, in 1899 (*23*). Chest radiography for mass screening and active case-finding for pulmonary TB was recommended by the New York City Health Department in the United States as early as the 1920s–1930s (*24*,*25*) and was widely implemented in industrialized countries in the 1940s–1960s, contributing to the success of TB control in developed countries (*3*,*26*). However, by the late 1960s, WHO no longer recommended active case finding through mass radiograph screening for developing countries because of financial constraints (*27*). Not until 2015 was chest radiograph screening re-recommended by WHO as part of the end TB strategy (*1*,*27*). Even today, radiograph screening remains inaccessible in resource-limited areas in many low- and middle-income countries. Screening criteria typically focus on previously underdiagnosed TB disease, which is known as the late stage of TB disease (*26*). As a consequence, the very early stage of TB, known as subclinical TB, which is characterized by minimal or atypical presentations on chest radiograph, could still be underrecognized. 

Nonetheless, our findings are consistent with several previous studies. In a study conducted in Czechoslovakia among a district-level cohort of 7,800 adults followed during 1961–1964 (*28*), 44% of persons in whom incident TB developed during follow-up had a previous abnormal chest radiograph indicating inactive TB or a fibrotic lesion. In a cohort study conducted during 1950–1952 in Denmark, persons with fibrotic lesions and shadows suspicious of TB on chest radiograph had a >10-fold higher annual rate of subsequent TB during the follow-up period (*29*). Similarly, in cohort studies conducted in 1988 among Southeast Asian refugees in Seattle (*30*) and in the early 1980s among recent Asian immigrants to northern Canada (*31*), persons with radiograph abnormalities indicative of or interpreted as inactive TB had 6-fold higher and 19-fold higher risk for subsequent TB. Those studies demonstrate that different types of lesions on baseline radiograph are associated with varying risks of subsequent TB in different mass screening settings. In a 1981 study from Hong Kong, 176 smear- and culture-negative adults with TB-compatible lesions were monitored for up to 30 months (*32*). Of those, TB was eventually diagnosed in 30%; most cases occurred within the first 12 months of follow-up. Similarly, a 1985 study conducted in South Africa found that among 152 TST-positive gold miners with evolving apical lung lesions on baseline radiograph who were smear- and culture-negative, bacteriologically confirmed pulmonary TB developed in 58% during a 58-month follow-up period (*33*). In a recent systematic review and meta-analysis of studies of 34 cohorts observed in the prechemotherapy era of persons with untreated TB who underwent follow-up, progression from microbiologically negative to positive disease was observed at an annualized rate of 10% in those with initial radiographs suggestive of active TB and at a rate of 1% in those initially suggestive of inactive TB (*12*). Although the incidence rate in this systemic review is lower than in our study, only 1 of the studies cited previously focused on a cohort of household contacts with known exposure to TB. The higher incidence in our study might be because we did not require microbiological confirmation to classify incident disease.

The finding that chest radiography might help identify pathological changes before the onset of TB symptoms aligns with recent evidence from South Africa. Esmail et al. (*34*) followed 250 HIV-negative adults exposed to TB at home for >4.7 years. Among 14 persons in whom active disease developed during follow-up, 6 exhibited baseline abnormalities on computed tomography/positron emission tomography (*34*). The TB research community now widely acknowledges that TB follows a dynamic continuum, encompassing TB infection, asymptomatic incipient or subclinical TB, and symptomatic clinically manifested disease (*7*–*10*). A 1982 study by the International Union Against Tuberculosis Committee on Prophylaxis revealed that among 28,000 adults with radiograph-identified fibrotic pulmonary lesions compatible with TB, a 24-week regimen of isoniazid preventive treatment decreased tuberculosis incidence by 65% compared with the placebo group over a 5-year follow-up period (*35*). Those findings suggest that lung changes can be observed in the asymptomatic phase of TB. Given that the cost of computed tomography/positron emission tomography makes it impractical for many screening programs, radiography could play a pivotal role in early TB detection, enabling early interventions such as close follow-up, preventive therapy, or full-dose combination therapy in populations at risk.

Our study revealed that young persons 16–24 years of age with abnormal baseline chest radiograph had a 3-fold higher risk for TB than older adults with abnormal radiograph. The observed association between the severity of baseline radiograph abnormalities and the trajectory of early TB disease within this young age group further supports the notion that an abnormal radiograph might serve as an indicator of undiagnosed or subclinical TB in this specific population, whereas older adults are more likely to have abnormal radiographs caused by non-TB lung diseases. That notion is supported by 2 cross-sectional studies conducted in Malawi during 2018–2020 (*36*) and the 2016 Kenya national TB prevalence survey (*37*). Both those studies reported a high prevalence of non-TB abnormalities on chest radiographs among older adults, with the proportion increasing with age.

The first limitation of this study is that only 40% of TST-positive adults received a baseline radiograph independent of symptoms; the decision to request a radiograph was made by local health center clinicians. Second, we lacked lateral positioning radiographs, which might have led to misclassifications of abnormalities. Third, readings were initially blinded for the first reader, and only abnormal radiographs were subsequently validated by a second reader who was not blinded. Therefore, misclassification of findings could have occurred. The fact that the second reader only read the films initially deemed to be abnormal and was not blinded to this classification might have resulted in overidentifying abnormalities in keeping with the first reader’s interpretations, leading to inflated measures of inter-rater agreement and consequently overestimating the effect size of the association between abnormalities and subsequent TB disease. Because the radiographs initially read as unremarkable were not read by a second reader, some of those might have been classified as abnormal if all radiographs had been read. Fourth, the relatively high prevalence of previous TB history among household contacts raises the possibility that some abnormal baseline radiographs were a result of inadequately treated previous TB or residual, minimally active TB. We did not have previous treatment or drug sensitivity information for those contacts and so cannot rule out the possibility that a history of TB might contribute to the elevated risk for incident TB among those persons. However, we also observed that persons with TB history were less likely to undergo baseline examinations. Moreover, when we excluded those with TB history from the 1,747 contacts who underwent radiograph screening, we found that the effect size increased by 30%. Finally, health center clinicians were expected to follow the clinical diagnosis guidelines outlined by Peru’s National TB Program, but different clinicians might have applied varying criteria for TB diagnosis. The absence of a standardized set of criteria for TB diagnosis might have resulted in nondifferential misclassification, potentially influencing the results toward a null effect.

Overall, our study revealed a significant association between abnormal baseline chest radiograph results and subsequent diagnosis of TB among asymptomatic adults with recent TB exposure. The severity of baseline radiograph results also reflected the early TB progression trajectory. Our findings strongly support the wide use of chest radiography with computer-aided detection as a screening tool in exposed adults and prompt the development of accurate criteria and algorithms for human and artificial intelligence readers to effectively identify early TB states beyond active TB disease, leading to timely intervention and proper management.

Appendix Additional information about chest radiograph screening for detection of subclinical tuberculosis in asymptomatic household contacts, Peru
