# Supplementary material for: Chest Radiograph Screening for Detecting Subclinical Tuberculosis in Asymptomatic Household Contacts, Peru
Source: Emerg Infect Dis. 2024 Jun;30(6):1115–24. doi: 10.3201/eid3006.231699 (PMC11138965; doi:10.3201/eid3006.231699)
Supplement: Appendix — Additional information about chest radiograph screening for detection of subclinical tuberculosis in asymptomatic household contacts, Peru [file 23-1699-Techapp-s1.pdf]

Article DOI: https://doi.org/10.3201/eid3006.231699

EID cannot ensure accessibility for supplementary materials supplied by authors. Readers who have difficulty accessing supplementary content should contact the authors for assistance.

Chest Radiograph Screening for Detecting Subclinical Tuberculosis in Asymptomatic Household Contacts, Peru

Appendix

CXR protocol

Definition of chest x-ray abnormal or not

'Abnormal' definition: the presence of any intrathoracic abnormalities suggestive of TB or not, including cavitation, non-cavitary parenchymal lesion, hilar lymphadenopathy, and pleural disorders on each chest radiograph (*1*–*3*).

Cavitation: A shining space that contains gas, measuring at least 1 cm in diameter inside the lung parenchyma, that is surrounded by an infiltrate or fibrotic wall with more than 1 mm in thickness.

Non-cavitary parenchymal lesion: Presence of the any features of parenchyma lesion that do not contain demonstrable cavitation, including patchy or confluent consolidation, ground glass opacity, non-calcified nodules, calcified nodules (Ghon focus), diffuse micronodules (miliary pattern), fibrosis, bronchiectasis, collapse (atelectasis), hyperinflation.

Hilar lymphadenopathy: Hilar and mediastinal lymph node (shadow) enlargement

Pleural disorders: Pleural effusion, pleural thickening, pleural calcification

E.g.,

A. Cavitation

B. Non-cavitary parenchymal lesion

C. Hilar lymphadenopathy

D. Pleural disorders

https://www.radiologymasterclass.co.uk/gallery/chest/pulmonary-disease/consolidation_lobar

Definition of Categories based on abnormal findings.

CXR findings suggestive of TB: presence of any upper lung zone predominant parenchymal lesions with or without cavitation, hilar lymphadenopathy, or pleural disorders compatible for adult-type TB (active and inactive) (*1*,*2*,*4*).

CXR findings nonsuggestive of TB: presence of any abnormalities inconsistent with the definition of CXR findings suggestive of TB.

Lung zones were determined by visualizing a perpendicular line from the apex of the lung to the hemidiaphragm and dividing the lung in half; the superior segment of the lower lobe was considered part of the upper lung zone (*2*,*3*,*5*). Descriptions of lesions associated with active and inactive tuberculosis, based on the 2008 US Department of Health and Human Services technical instructions for medical examinations (*1*).

Definition of chest x-ray severity-grad method

To grade the extent of abnormalities in CXR, percentage of assumed affected lung by any pathology was reported for each of three zones (upper, middle, or lower zones) in each lung (grade 0-1). The total percentage of lung affected was estimated by the sum of the percentage of six zones (grade 0-6), based on an established approach of Chest X ray score (Timika score) for grading CXR severity in adults (*6*).

Sum of the area (0%, 25%, 50%, 75%, 100%) of abnormal image of 6 regions

E.g., 0.25 + 0 + 0.5 + 0 + 0 + 0 = 0.75

References

1. US Department of Health & Human Services. Technical instructions for civil surgeons; tuberculosis medical examination. May 2008 [cited 2023 Mar 6]. https://stacks.cdc.gov/view/cdc/22142

2. Ravenel JG, Chung JH, Ackman JB, de Groot PM, Johnson GB, Jokerst C, et al.; Expert Panel on Thoracic Imaging. ACR Appropriateness Criteria imaging of possible tuberculosis. J Am Coll Radiol. 2017;14(5S):S160–5. PubMed https://doi.org/10.1016/j.jacr.2017.02.022

3. Lau A, Lin C, Barrie J, Winter C, Armstrong G, Egedahl ML, et al. The radiographic and mycobacteriologic correlates of subclinical pulmonary TB in Canada: a retrospective cohort study. Chest. 2022;162:309–20. PubMed https://doi.org/10.1016/j.chest.2022.01.047

4. Wetscherek MTA, Sadler TJ, Lee JYJ, Karia S, Babar JL. Active pulmonary tuberculosis: something old, something new, something borrowed, something blue. Insights Imaging. 2022;13:3. PubMed https://doi.org/10.1186/s13244-021-01138-8

5. Lau A, Lin C, Barrie J, Winter C, Armstrong G, Egedahl ML, et al. A comparison of the chest radiographic and computed tomographic features of subclinical pulmonary tuberculosis. Sci Rep. 2022;12:16567. PubMed https://doi.org/10.1038/s41598-022-21016-7

6. Ralph AP, Ardian M, Wiguna A, Maguire GP, Becker NG, Drogumuller G, et al. A simple, valid, numerical score for grading chest x-ray severity in adult smear-positive pulmonary tuberculosis. Thorax. 2010;65:863–9. PubMed https://doi.org/10.1136/thx.2010.136242

Appendix Table 1. Clinical Characteristics of HHCs Receiving Baseline CXR and Those Who Did Not*

Clinical characteristics (n = 4,506)	HHCs with baseline CXR (No. = 1,848)	HHCs without baseline CXR (No. = 2,658)	X^2 p value
Age (years) n (%)			
15–25	399 (22)	586 (22)	0.12
25–44	744 (40)	1135 (43)	
>45	705 (38)	937 (35)	
Male n (%)	684 (37)	1198 (45)	0.001
HIV+	7 (0.4)	20 (0.8)	0.02
Diabetes	51 (3)	80 (3)	0.34
BMI< = 18.5	14 (0.8)	8 (0.3)	0.52
BMI>30	423 (23)	550 (21)	0.08
Previous TB history	322 (17)	695 (26)	<0.001
With BCG scars	1,672 (90)	2,398 (90)	0.69
Heavy Smoker	79 (4)	131 (5)	0.54
Heavy Drinker	157 (8)	246 (9)	0.40
Any TB symptoms†	358 (19)	535 (20)	0.54

*BCG, Bacillus Calmette–Guérin; BMI, body mass index; CXR,chest x-ray; HHC, household contact; TB, tuberculosis.
†Presence of any of TB relevant symptoms including cough>14 days, coughing blood or phlegm, fever, shortness of breath, or night-sweats.

Appendix Table 2. Parameters of Chest Radiograph Evaluation*

Parameter	Evaluation
Parameter 1†	1) Being abnormal; 2) Being normal
Parameter 2 (abnormal CXRs)‡	1) Abnormalities suggestive of TB; 2) Abnormalities not suggestive of TB
Parameter 3 (abnormal CXRs)‡	The extent of abnormalities

*CXR, chest x-ray; TB, tuberculosis.
†The baseline abnormal CXRs were validated by 2 readers (n=135).
‡The baseline abnormal CXRs of the household contacts who subsequently developed TB disease were validated by 2 readers (n=27).

Appendix Table 3. Baseline CXR Findings for Each Type and Risk of Incident TB*

CXR abnormalities (N = 135)	HHCs who developed TB disease (n = 27)	HHCs who did not develop TB disease (n = 108)
Cavities	2 (7%)	5 (5%)
Non-cavitary parenchymal lesions†	18 (67%)	100 (92%)
Hilar lymphadenopathy	6 (22%)	6 (5%)
Pleural disorders	4 (15%)	11(10%)
Suggestive of TB‡	14 (52%)	44 (41%)

*CXR, chest x-ray; HHC, household contact;TB, tuberculosis.
†Presence of the any features of parenchyma lesion that do not contain demonstrable cavitation, including patchy or confluent consolidation, ground glass opacity, non-calcified nodules, calcified nodules (Ghon focus), diffuse micronodules (miliary pattern), fibrosis, bronchiectasis, collapse (atelectasis), hyperinflation.
‡The TB suggestive category among the baseline abnormal CXRs from the 135 HHCs was determined by one reader. Of these, the CXRs of 27 HHCs who developed TB disease were validated by the second reader.

Appendix Table 4. The risk of incident TB among the household contacts with abnormal baseline CXRs and the agreement between 2 readers in interpreting their CXRs*

Agreement	HHCs who did not develop TB disease (n = 108)	HHCs who developed TB disease (n = 27)	Total number	X^2 p value
Was the CXR abnormal? (n = 135)				0.32
Agree	107 (99%)	26 (97%)	133 (99%)	
Disagree	1 (1%)	1(3%)	2 (1)	
Did the CXR show cavitation? (n = 135)				1.00
Agree	102 (94%)	26 (96%)	128 (95%)	
Disagree	6 (6%)	1 (4%)	7 (5%)	
Did the CXR show non-cavitary parenchymal lesions? (n = 135)				0.004
Agree	102(94%)	20 (74%)	122 (90%)	
Disagree	6 (6%)	7 (26%)	13 (10%)	
Did the CXR show hilar lymphadenopathy? (n = 135)				0.49
Agree	106 (98%)	26(96%)	132 (98%)	
Disagree	2 (2%)	1(4%)	3 (2%)	
Did the CXR show pleural abnormalities? (n = 135)				0.26
Agree	105 (97%)	25 (93%)	130 (96%)	
Disagree	3 (3%)	2 (7%)	5 (4%)	

*CXR, chest x-ray; HHC, household contact; TB, tuberculosis.

Appendix Table 5. Different age groups and their association with risk of subsequent TB in general subjects (N = 1,747, incident events = 52)

		Univariate (n=1,747, events=52)		Multivariate‡ (n=1,630, events=49)	
Age group, y†	Incident TB (%)	Hazard Ratio (95% CI)	p value	Hazard Ratio (95% CI)	p value
Age ≥45	14 (2%)	ref	—	ref	—
Age 25–44	13 (2%)	0.87 (0.41–1.86)	0.73	0.96 (0.44–2.08)	0.91
Age 16–24	25 (7%)	3.29 (1.71–6.34)	<0.001	2.40 (1.11–5.17)	0.03

*CXR, chest x-ray; HHC, household contact; TB, tuberculosis.
†HHCs with an abnormal CXR at baseline were categorized into three age groups: group of Age ≥45, n=668; group of Age 25–44, n=709; group of Age 16–24, n=370.
‡Adjusted for symptoms and CXR screening results, age, sex, alcohol use, tobacco use, diabetes, hypertension, cardiovascular disease, kidney disease, asthma, previous TB history and body mass index. HIV-positive subjects (n=4) were excluded.

Appendix Table 6. Different age groups and their association with risk of subsequent TB in adults with an abnormal CXR at baseline (N = 135, incident events = 27)*

Age group (years)†	Incident TB N (%)	Univariate (n = 135, events = 27) Hazard Ratio (95% CIs)	Univariate P-value	Multivariate‡ (n = 128, events = 26) Hazard Ratio (95% CIs)	Multivariate P-value
Age >45	9 (14%)	ref	—	ref	—
Age 25–44	6 (13%)	0.99 (0.35–2.77)	0.98	0.95 (0.31–2.96)	0.93
Age 16–24	12 (50%)	4.61 (1.94–10.96)	<0.001	3.52 (1.16–10.65)	0.03

*CXR, chest x-ray; HHC, household contact; TB, tuberculosis.
†HHCs with an abnormal CXR at baseline were categorized into three age groups: group of Age ≥ 45, n=66; group of Age 25–44, n=45; group of Age 16–24, n=24.
‡Adjusted for symptoms, age, sex, alcohol use, tobacco use, diabetes, hypertension, cardiovascular disease, kidney disease, asthma, previous TB history and BMI. All of the 135 subjects were HIV-negative.

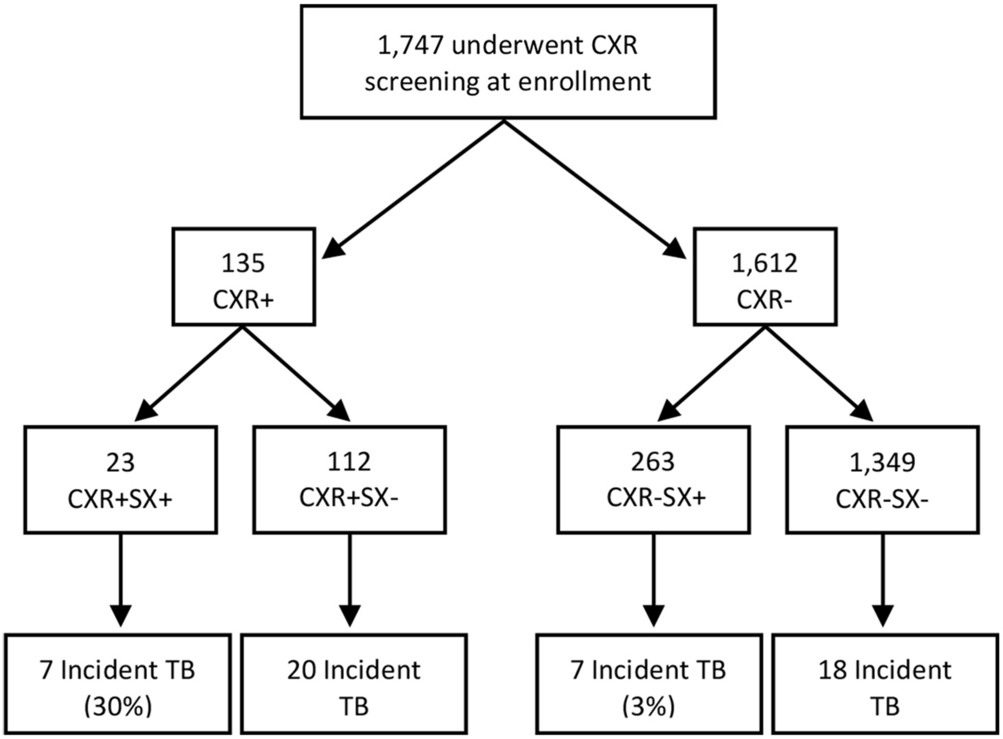

Appendix Figure 1. Flowchart of 1,747 TST positive adult contacts who underwent both symptom and radiography screening at enrollment. SX-, no symptoms; SX+, symptoms; CXR-, normal CXR, CXR+, abnormal CXR.

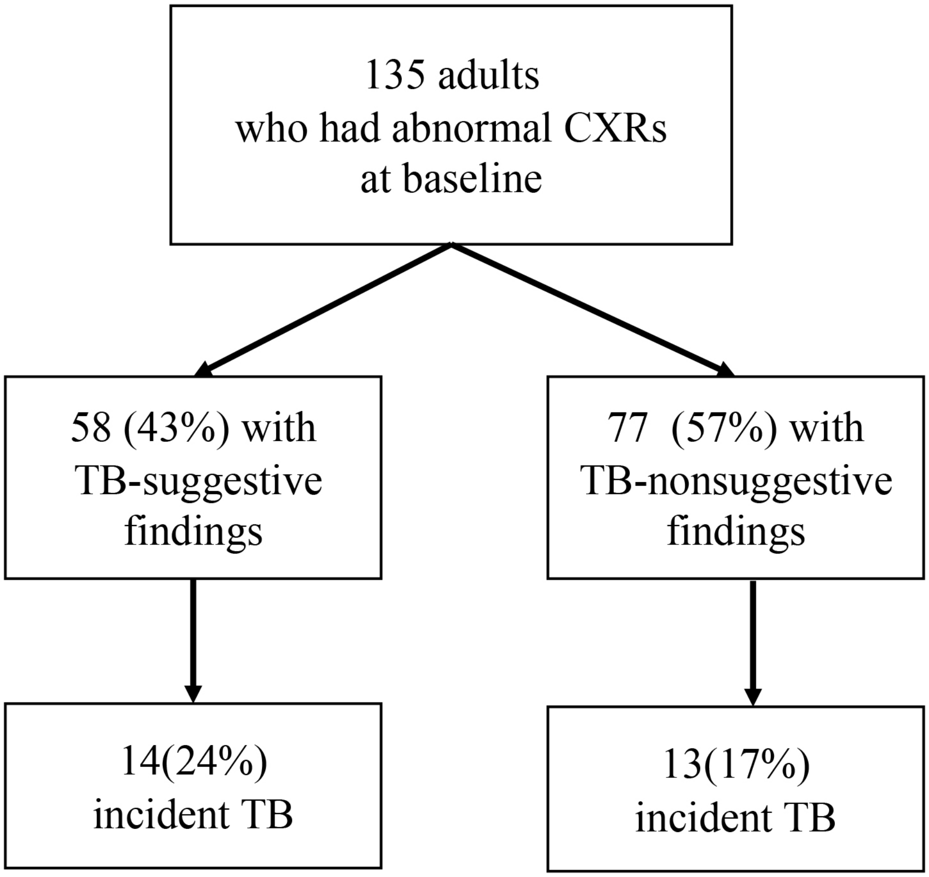

Appendix Figure 2. The abnormal CXR findings and risk of developing incident TB among adults 135 subjects with an abnormal baseline CXR. *Appendix Figure S3*. Age distribution of enrolled household contacts (age>15 years, n = 1,747).

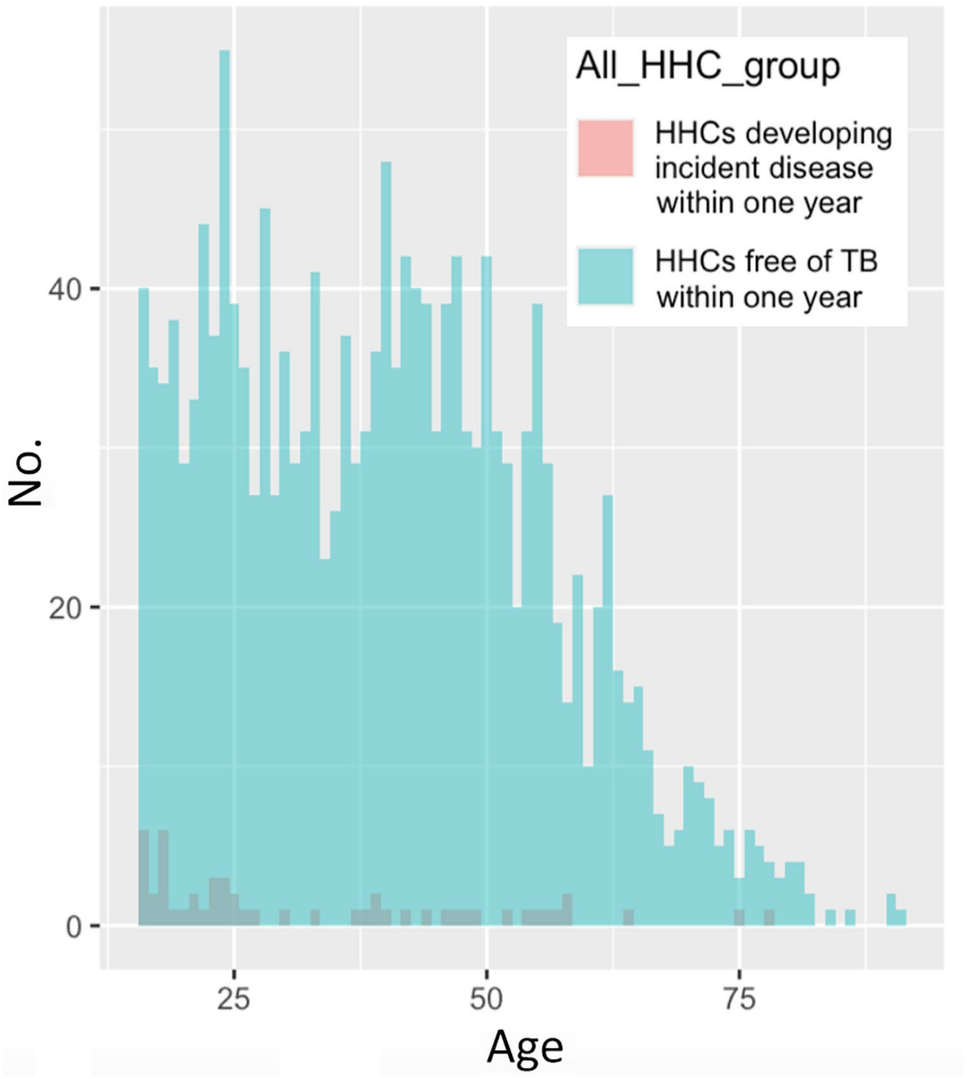

Appendix Figure 3. Age distribution of enrolled household contacts (age>15 years, n = 1,747)

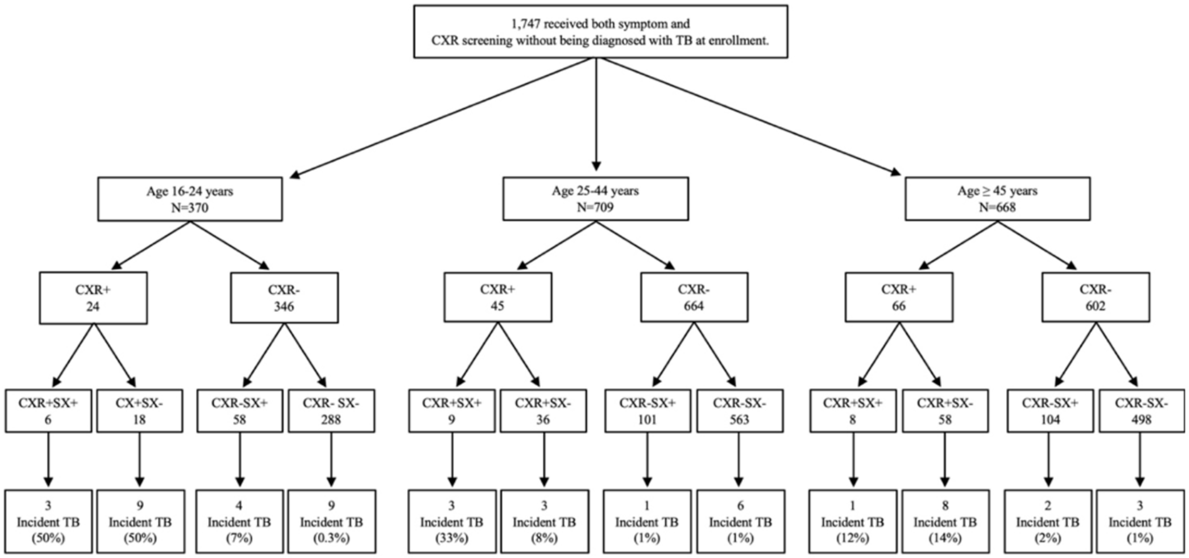

Appendix Figure 4. Flow-chart of 1,747 TST positive adult contacts who underwent both symptom and radiography screening at enrollment stratified by age groups. SX-, SX-, no symptoms; SX+, symptoms; CXR-, normal CXR, CXR+, abnormal CXR.

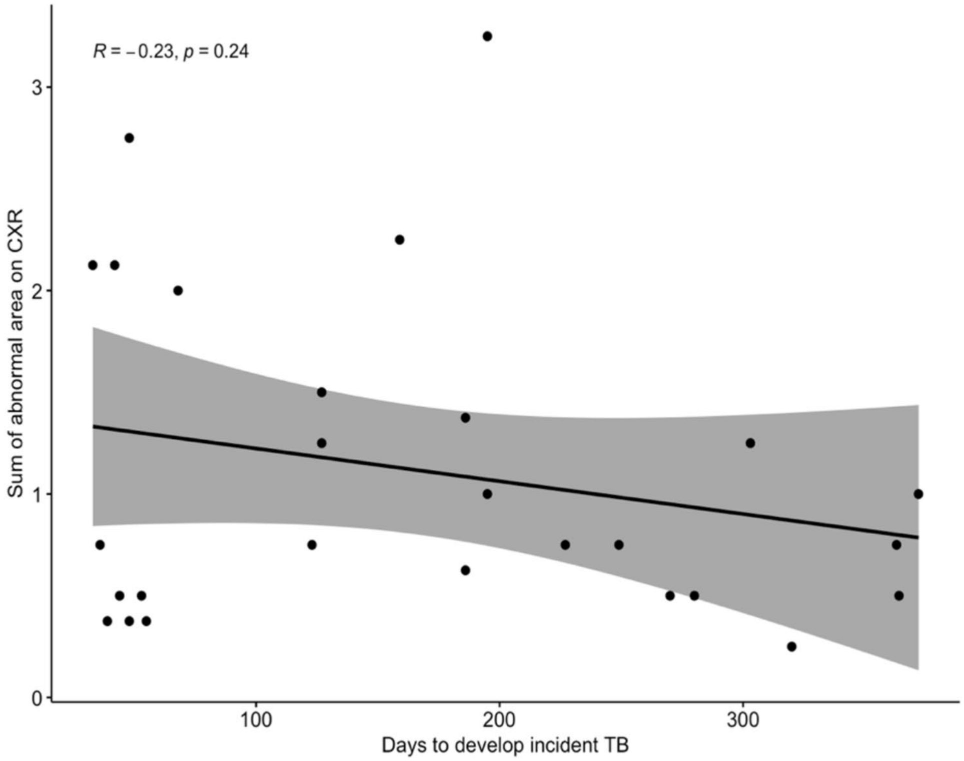

Appendix Figure 5. The association between degree of baseline CXR severity and time to developing incident TB among subjects with abnormal CXR findings (n = 27). Mean difference in days [95% CIs] = −0.002[-0.005 – 0.001], n = 27.

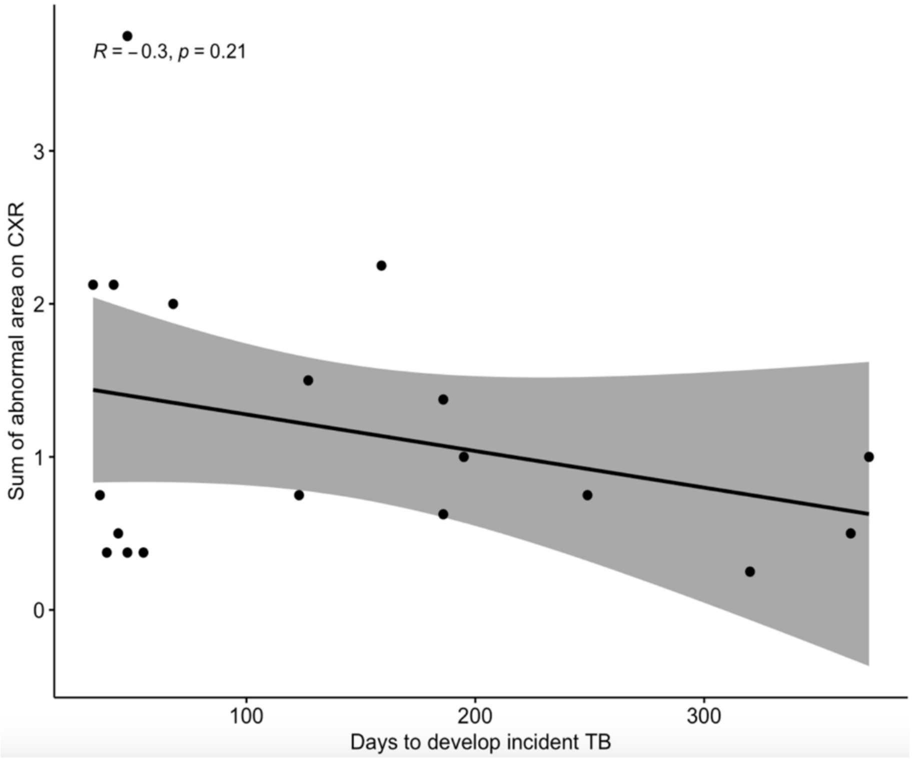

Appendix Figure 6. The association between degree of baseline CXR severity and time to developing incident TB among subjects with abnormal CXR findings who had no previous TB history (n = 19). Mean difference in days [95% CIs] = −0.002[-0.006 – 0.001], n = 19.
